# CCR5-targeted γδ CAR-T cells: a rational step toward HIV-resistant allogeneic immunotherapy

**DOI:** 10.3389/fphar.2025.1734423

**Published:** 2025-12-02

**Authors:** Lina Hu, Haiyan Zhou

**Affiliations:** Department of Infectious Diseases, Weifang People’s Hospital, Weifang, Shandong, China

**Keywords:** γδ CAR-T cells, CCR5 disruption, HIV resistance, allogeneic immunotherapy, B-cell malignancies

## Introduction

Adoptive T-cell therapy has transformed the treatment landscape of several hematologic malignancies. However, patients living with HIV infection have been largely excluded from this progress. Autologous CAR-T-cell manufacturing faces unique obstacles in this population, including low T-cell counts, impaired T-cell fitness, and the risk of HIV infection of the engineered cells themselves. In a recent publication in Nature Biomedical Engineering, Ramírez-Fernández et al. (2025) introduced a technically sound and biologically informed strategy to address this gap: engineering γδ T cells with a CD19 CAR integrated at the CCR5 locus, thereby simultaneously generating an allogeneic cell product and conferring HIV resistance (Ramírez-Fernández et al., 2025).

γδ T cells constitute a small but functionally diverse subset bridging innate and adaptive immunity. They recognize antigens independently of MHC through stress-induced ligands and butyrophilin family molecules rather than peptide–MHC complexes. Two principal human subsets—Vγ9Vδ2 T cells, predominant in peripheral blood and reactive to phosphoantigens, and Vδ1 T cells, enriched in tissues and recognizing non-peptidic stress ligands—exhibit distinct effector phenotypes and cytokine profiles (Hu et al., 2023). These characteristics, together with their low graft-versus-host potential, make γδ T cells particularly attractive for “off-the-shelf” CAR platforms and for treating immunocompromised or virally infected hosts.

Nevertheless, clinical translation of γδ CAR-T therapies has been constrained by low baseline abundance, inefficient gene editing, and differentiation during expansion (Kabelitz et al., 2020). Ramírez-Fernández and colleagues combined γδ T-cell expansion using artificial antigen-presenting cells (aAPCs) with CRISPR/Cas9-mediated knock-in of a CD19 CAR at the CCR5 locus, thereby simultaneously introducing CAR expression and disrupting a major HIV co-receptor. This dual engineering strategy reflects a rational alignment of biological mechanism with clinical context.

## Methodological advances

The central innovation of this study lies in integrating CAR engineering and HIV resistance in a single genetic modification. Using CRISPR/Cas9 and rAAV donor templates, the authors precisely targeted the CCR5 locus to insert the CD19 CAR. CCR5 serves as a co-receptor for HIV entry, and its disruption has long been associated with protection against CCR5-tropic HIV strains-a principle derived from the well-known “Berlin patient” case and subsequent gene editing strategies in T cells (Rothemejer et al., 2023). By placing the CAR-Transgene in this locus, the researchers achieved both stable CAR expression and resistance to HIV infection in engineered γδ T cells.

The study employed artificial antigen-presenting cells (aAPCs) to stimulate robust γδ T-cell proliferation while maintaining a central and effector memory phenotype. Transcriptomic and phenotypic analyses confirmed that the expanded cells were not terminally differentiated or exhausted-a key factor for *in vivo* persistence. This step addresses a crucial technical hurdle in γδ CAR-T development: generating a sufficient yield of high-quality cells suitable for therapeutic use (Kabelitz et al., 2020; Su et al., 2025).

Functionally, the resulting γδ CCR5KI-CAR19 cells exhibited potent cytotoxicity against CD19^+^ targets *in vitro*. Furthermore, they demonstrated marked resistance to HIV infection and depletion compared to control γδ T cells, validating the functional impact of CCR5 disruption. These findings are carefully framed as preclinical, without claims of clinical efficacy, but they provide a credible mechanistic foundation.

*In vivo* activity was evaluated using two complementary models: the chick chorioallantoic membrane (CAM) assay and NSG xenografts bearing NALM6 leukemia or lymphoma. In both models, γδ CCR5KI-CAR19 cells effectively suppressed tumor burden relative to controls. These models are immunodeficient and cannot recapitulate the full complexity of human immunity, but they offer proof of principle that the engineered cells retain anti-tumor activity in a living host environment.

## Relevant advances in the field

This work builds on multiple research threads in tumor immunology, cell therapy, and HIV resistance. First, γδ T-cell biology has gained renewed attention over the past decade. γδ T cells display unique stress-sensing mechanisms and broad tumor reactivity, particularly γ9δ2 T cells, which recognize phosphoantigens through butyrophilin molecules (Kabelitz et al., 2020). Early clinical studies of γδ T-cell transfer demonstrated safety but limited efficacy, in part due to manufacturing and persistence challenges. However, advances in expansion protocols and gene editing have reinvigorated this field.

Second, CCR5 targeting has a long history in HIV therapeutics. Disruption of CCR5 confers resistance to CCR5-tropic HIV strains, and the “Berlin patient” provided proof of concept that CCR5 deletion can enable long-term viral control. Since then, multiple groups have used gene editing approaches, including zinc finger nucleases, TALENs, and CRISPR, to inactivate CCR5 in T cells and hematopoietic progenitors (Rothemejer et al., 2023). Ramírez-Fernández et al. extend this concept by integrating CCR5 disruption directly into CAR-T engineering, ensuring HIV resistance without requiring additional edits.

Finally, the field is moving toward allogeneic CAR-T platforms as a scalable alternative to autologous manufacturing. Approaches include TCR knockout in αβ T cells, HLA editing, and the use of alternative effector cells such as NK or γδ T cells (Su et al., 2025; Azeez et al., 2025). γδ T cells are particularly promising because they naturally circumvent HLA restrictions, reducing the need for extensive genome engineering. Previous studies established proof-of-principle for γδ CAR-T-cell activity, but the integration of HIV resistance through CCR5 targeting represents a meaningful conceptual advance.

Recent studies have advanced γδ CAR-T research toward clinical translation. Vδ1-based CAR-T products have entered early-phase trials for hematologic and solid malignancies, demonstrating encouraging safety and preliminary efficacy (Makkouk et al., 2021; Neelapu et al., 2022). Other approaches combine γδ CAR-T cells with checkpoint blockade or cytokine support (e.g., IL-15) to enhance persistence and trafficking (Ferry et al., 2022). These efforts collectively highlight the growing clinical interest in γδ cell platforms as allogeneic immunotherapy candidates.

## Strengths and limitations

A major strength of the study lies in its alignment between target choice and clinical need. CCR5 targeting simultaneously supports stable CAR integration and confers protection from HIV, directly addressing the vulnerabilities of the intended patient population. The aAPC expansion method generates cells with desirable memory-associated phenotypes, which are believed to correlate with better persistence and anti-tumor activity. Moreover, the experimental evidence is methodically presented, with clear distinctions between *in vitro* mechanistic data and preclinical efficacy readouts.

The *in vitro* data demonstrate on-target CD19 killing and HIV resistance; the *in vivo* models confirm tumor control in immunodeficient systems. This multi-layered approach mirrors the stepwise progression often seen in successful translational platforms. Importantly, the authors refrain from overinterpreting their results. Rather than making claims of clinical benefit, they frame the work as a technical and biological foundation for future development.

Still, there are clear limitations. The study was conducted entirely in preclinical settings, and no data exist on persistence, trafficking, or efficacy in immunocompetent or HIV-positive hosts. NSG xenografts and CAM assays cannot model host immune responses or viral dynamics. Allogeneic safety remains untested clinically. Furthermore, CCR5 disruption protects against CCR5-tropic HIV but not necessarily other strains, such as X4-tropic or dual-tropic variants. These are important caveats for future translation.

## Translational outlook

This work outlines a plausible pathway toward expanding CAR-T therapy to a historically underserved population. Patients living with HIV and B-cell malignancies face dual challenges: limited treatment options and exclusion from many immunotherapy trials. An allogeneic, HIV-resistant γδ CAR-T platform could overcome many logistical and biological barriers that prevent these patients from accessing autologous products.

Beyond HIV, this study contributes to the broader landscape of allogeneic immunotherapy. γδ T cells could be engineered to target other tumor antigens or combined with additional edits to improve persistence and overcome immunosuppressive signals. CCR5 is not the only possible engineering site, but it represents a rational and clinically meaningful choice for the initial proof of concept.

The manufacturing strategy employed here is based on established technologies, including feeder expansion and CRISPR knock-in, positioning it well for GMP translation. If these results can be reproduced under clinical-grade conditions, early-phase trials focused on safety, persistence, and antiviral resistance would be justified.

## Discussion

This study represents a technically rigorous and conceptually coherent advance in cellular immunotherapy. Rather than overpromising therapeutic benefit, the authors focus on solving a practical bottleneck—how to engineer γδ CAR-T cells that are both functional against cancer and resistant to HIV infection. This approach reflects a broader shift in cell therapy development toward rational design strategies that align molecular engineering with disease biology.

By targeting CCR5, the authors leverage a well-characterized vulnerability in HIV pathogenesis. By choosing γδ T cells, they circumvent many of the barriers that complicate allogeneic αβ CAR-T development. And by focusing on robust, well-defined preclinical readouts, they lay the groundwork for future clinical investigation without overstating conclusions.

The next steps are clear: clinical-scale manufacturing, testing in humanized HIV-tumor co-models, formal assessment of allogeneic safety, and early-phase clinical trials. If these efforts succeed, the platform could meaningfully expand access to CAR-T therapy and establish γδ cells as a competitive backbone for universal immunotherapy strategies.

## Future directions and clinical perspective

The CCR5-targeted γδ CAR-T concept represents a rational foundation for HIV-resistant allogeneic cell therapy. Next steps should emphasize GMP-compliant manufacturing, evaluation in humanized HIV–tumor models, and formal assessment of long-term persistence and safety. Clinically, such platforms could extend CAR-T accessibility to people living with HIV and inform broader allogeneic strategies for infection-associated or immunodeficient malignancies.
